# Specialized Mental Health Crisis Response Activities Within US Law Enforcement Agencies

**DOI:** 10.1007/s10597-025-01507-3

**Published:** 2025-08-04

**Authors:** Zoe Lindenfeld, Amanda I. Mauri, Saba Rouhani, Charley E. Willison

**Affiliations:** 1 Edward J. Bloustein School of Planning and Public Policy, Rutgers University, New Brunswick, NJ 08901 USA; 2 Department of Health Policy and Management, School of Public Health, University of Maryland, College Park, MD USA; 3 Department of Public Health Policy and Management, School of Global Public Health, New York University, 726 Broadway, New York, NY USA; 4 Department of Public and Ecosystem Health, Cornell University, Ithaca, NY USA

**Keywords:** Law enforcement, Mental health, Crisis, Behavioral health, Police

## Abstract

**Objective:**

This study examines the prevalence of specialized police responses to persons experiencing a mental health crisis across U.S. law enforcement agencies and explores whether organizational and community factors are associated with their presence.

**Methods:**

This study used 2020 data from a nationally representative survey of over 2,500 law enforcement agencies. The primary outcomes included whether agencies implemented one of four responses: (1) designated unit, (2) designated personnel, (3) addressed mental health without designated unit or personnel, or (4) did not address. Logistic regression models assessed factors associated with each response type.

**Results:**

Over half (51.0%, *n* = 1,349) of agencies addressed mental health but lacked designated units or personnel, while 6.9% (*n* = 183) did not specifically address mental health. Larger agencies, agencies located in urban areas, as well as those with external partnerships, and a higher number of use of force complaints were significantly more likely to designate a unit or personnel.

**Conclusions:**

Fewer than half of law enforcement agencies have responses for mental health crises. Further research is needed to identify barriers and facilitators to adopting specialized responses, particularly among rural and under-resourced agencies.

## Introduction

In the United States, individuals with serious and persistent mental illness disproportionately encounter the police, with an estimated 7-31% of all police calls involving a person exhibiting signs of a mental health disorder (Abramson, 2021; Shapiro et al., 2015). These interactions place persons with serious mental illness at increased risk of police harm; those displaying signs of mental illness are over seven times more likely to be killed in police shootings compared to others (Marcus & Stergiopoulos, 2022; Saleh et al., 2018). Further, initial encounters with law enforcement can trigger a cascade of additional involvement with the criminal legal system, contributing to the overrepresentation of individuals with mental illness in prisons and jails (Dumont et al., 2012; Steadman et al., 2000). Moreover, prior involvement in the criminal legal system increases the risk of being unhoused, unemployed, uninsured, and re-incarcerated, perpetuating a cycle of criminal legal entanglement and further marginalization (Baillargeon et al., 2009; Wildeman & Wang, 2017).

Given the overrepresentation of persons with mental illness in police encounters, and to mitigate harms to this population, law enforcement agencies across the country have adopted various strategies to minimize harmful interactions between the police and persons experiencing a mental health crisis. While numerous programs focus on training officers to respond to mental health crises—such as Crisis Intervention Team (CIT) training—departments implement these trainings in diverse ways (Steadman et al., 2000). Some agencies have invested substantial resources in creating specialized units to respond to mental health-related service calls. These units are staffed with officers trained to identify individuals experiencing a mental health crisis, de-escalate situations, and connect individuals to follow-up care outside of the criminal justice system (Shapiro et al., 2015; Watson et al., 2021). These units may be police-only, or take on a co-response form in which police respond alongside a behavioral health clinician (Balfour et al., 2022; SAMHSA, 2025.; Shapiro et al., 2015). Others offer mental health training to some officers but do not establish dedicated units, instead requiring trained officers to continue their regular patrol duties. And some agencies do not offer formal mental health training to any or all of their officers (Shapiro et al., 2015; Watson et al., 2021).

While prior studies have examined the implementation of specific models, such as the CIT model or co-response models, in limited samples of law enforcement agencies (Compton et al., 2006; Deane et al., 1999; Hails & Borum, 2003), to date, no research has examined the prevalence of different specialized police responses to mental health crises nationwide, or how community and organizational characteristics may influence the adoption of these approaches. To address this gap, we analyze data from a nationally representative survey of over 2,500 law enforcement agencies. Specifically, we assess the proportion of agencies that have implemented specialized mental health crisis response activities, using a survey question which categorizes these activities along a continuum from no specialized response to the major reform of establishing a specialized mental health crisis response unit. In addition, we explore organizational and county-level factors associated with the adoption of varying degrees of police mental health crisis reforms. At the organizational level, we prioritize variables that have been previously empirically linked to greater adoption of new policies and programs within police agencies, including larger department size and operating budget, having partnerships with community members (Nowacki & and Willits, 2018; Teti, 2024), and more technological expertise (Skogan & Hartnett, 2005). Additionally, given that community pressure has been linked to organizational innovation within police departments, we include a variable corresponding to the number of use of force complaints in a department, operationalized as a per capita measure standardized by the number of sworn officers in the department, to account for variation in department size. (Katz, 2001). At the county level, given that law enforcement responses may impact communities with higher numbers of racial minorities differently, we include measures reflecting the demographic characteristics of the counties service by law enforcement agencies (Kahn & Martin, 2016).

As federal, state, and local governments increasingly invest in reforms aimed at transforming police responses to mental health crises (Centers for Medicare & Medicaid Services (CMS), 2025; Substance Use and Mental Health Services Administration (SAMHSA), 2020), our findings provide critical insights into the variation in police approaches and whether reforms concentrate in communities with specific organizational and community characteristics. Specifically, this study offers a timely overview of mental health response models in US. law enforcement agencies as of 2020, serving as critical baseline data for assessing subsequent developments in the field, particularly those catalyzed by the 2020 murder of George Floyd and the expanded investment in alternative crisis response models (Saunders et al., 2023). Understanding heterogeneity in local police responses to mental illness is of further, increasing importance in the face of uncertain federal resources to support reforms.

## Methods

### Data

This study uses data from the Bureau of Justice Statistics’ (BJS) Law Enforcement Management and Administrative Statistics (LEMAS) survey, which collects data on a range of topics from a nationally representative sample of over 3,000 general-purpose, county, and local law enforcement agencies (BJS, 2025). The survey is completed by agency staff members designated by the agency’s chief executive (*Law Enforcement Core Statistics*, 2020). The LEMAS is conducted every four years, and the current analysis utilized data from the 2020 survey, the most recent available. As part of the survey, the LEMAS asks law enforcement agencies: “How did the agency address this problem/task: Mental health.” The mutually exclusive response options for this question constitutes our four main dependent variables: (1) The agency had specialized units to address mental health; (2) The agency has designated personnel but no specialized unit to address mental health; (3) The agency addresses mental health but has no designated units or personnel; and (4) The agency does not address formally mental health.

We extracted several independent variables from the LEMAS survey. Most variables were directly extracted from survey questions, which included predefined response options. This included data on geographic location, agency type (sheriff vs. local/county), agency size (number of sworn officers; operating budget per capita, calculating using county population estimates from the American Community Survey), partnerships with advocacy organizations, the number of use of force complaints received annually (operationalized as a per capita measure by number of officers), and whether officers wear body cameras while on patrol. Appendix Table 3. reports all survey questions used in this analysis.

**Table 1:** Descriptive statistics of US law enforcement agencies by mental health crisis response activities, 2020 (*n* = 2,646)

Variable	Agency had specialized unit to address mental health	Agency had designated personnel to address mental health	Addressed mental health, no unit/personnel	Agency did not address mental health	Total
N	380 (14.4%)	734 (27.7%)	1,349 (51.0%)	183 (6.9%)	2,646 (100.0%)
Agency type					
*Sheriff*	96 (25.3%)	179 (24.4%)	269 (19.9%)	29 (15.8%)	573 (21.7%)
*Local/county police*	284 (74.7%)	555 (75.6%)	1,080 (80.6%)	154 (84.2%)	2,073 (78.3%)
Agency partners with advocacy organizations	285 (75.0%)	478 (65.3%)	628 (46.9%)	41 (22.7%)	1,432 (54.4%)
Operating budget per capita (# county residents)^Ta^	142.39 (215.92) [103.51]	93.52 (139.39) [51.01]	75.13 (285.72) [21.55]	42.70 (95.76) [6.99]	87.83 (234.58) [36.50]
Number of full-time sworn officers^T a^	567.823 (2,103.653) [172.0]	131.620 (250.699) [64.50]	53.736 (211.003) [15.0]	27.577 (80.205) [6.0]	149.211 (847.654) [26.0]
# of use of force complaints (per capita)^T^	0.04 (0.22)	0.02 (0.11)	0.01 (0.07)	0.03 (0.13)	0.02 (0.12)
Officers wear body cameras	278 (73.5%)	487 (67.1%)	835 (61.9%)	112 (61.2%)	1,712 (65.0%)
Percent Black in agency’s county^T^	12.794 (14.518)	11.691 (12.620)	9.428 (12.147)	12.517 (15.910)	10.754 (12.997)
Percent Hispanic in agency’s county^T^	18.906 (16.558)	14.134 (16.666)	11.050 (13.447)	8.773 (9.985)	12.878 (14.961)
Percent of households with income below the federal poverty limit in agency’s county^T^	8.90 (4.07)	9.24 (4.32)	9.39 (4.41)	10.78 (5.63)	9.38 (4.45)
Agency located in rural county	43 (11.3%)	182 (24.8%)	577 (42.8%)	90 (49.2%)	892 (33.7%)
Region					
*Northeast*	44 (11.58)	133 (18.19)	246 (18.28)	28 (15.30)	451 (17.08)
*South*	136 (35.79)	284 (38.85)	494 (36.70)	84 (45.90)	998 (37.80)
*Midwest*	88 (23.16)	215 (29.41)	455 (33.80)	61 (33.33)	819 (31.02)
*West*	112 (29.47)	99 (13.54)	151 (11.22)	10 (5.46)	372 (14.09)

^T^Reports mean and SD in parentheses

^a^Median value reported in brackets

We also extracted several county-level measures corresponding to the percentage of county residents who are Black, Hispanic, and households with income below the federal poverty limit from the (Shapiro, et al., 2020) American Community Survey 5-year estimates (USCB, 2025). We included an indicator for whether an agency is located in a rural county from the US Department of Agriculture Rural-Urban Continuum Codes (U.S Department of Agriculture, 2025), as well as an indicator for geographical Region of the US (Northeast, South, Midwest, West).

#### Analysis

The full LEMAS survey includes 3,499 law enforcement agencies. Observations were dropped from the sample if missing data on our outcome of interest (*n* = 805), or if the agency was a state law enforcement agency (*n* = 48 observations). We excluded state police agencies given that these forces may have authority limited to specific activities, such as state highway patrol, and have less authority to perform general law enforcement duties (Reisig & Correia, 1997). Descriptive statistics entailed taking the mean for the agency-level and county-level variables for all agencies and the four groups reflecting our dependent variable: agencies with specific units to address mental health, agencies with designated personnel to address mental health, agencies that addressed mental health but did not have specific units or designated personnel, and agencies that did not address mental health. We then conducted four cross-sectional logistic regression models with different mental health crisis response activities as our dependent variable (model 1: agency had specialized unit to address mental health; model 2: agency had designated personnel to address mental health; model 3: agency addressed mental health but had no unit or personnel; model 4: agency did not formally address mental health). Each model adjusted for organizational and county-level characteristics, as well as US region. We accounted for clustering of police agencies within states by using state-clustered standard errors to adjust for within-state correlation in the logistic regression models. All analyses were conducted with Stata SE 18. The Rutgers University Institutional Review Board deemed this study exempt from review. We followed the Strengthening the Reporting of Observational Studies in Epidemiology (STROBE) reporting guideline.

## Results

Table 1. provides organizational and county-level characteristics of the police agencies (*n* = 2,646) included in our study, organized by mental health crisis response model. Of these agencies, 14.4% (*n* = 380) had implemented a specialized unit to address mental health, 27.7% (*n* = 734) had designated personnel to address mental health, 51.0% (*n* = 1,349) reported that they addressed mental health but did not designate any specific units or personnel for this purpose, and 6.9% (*n* = 183) reported not specifically addressing mental health. Compared to agencies with other response models, agencies with specialized units to address mental health had the highest percentage of agencies that partnered with advocacy organizations (75.0%, *n* = 285), and agencies in which officers wear body cameras (73.5%, *n* = 278). Agencies with specialized units also had the highest mean number of full-time sworn officers (567.82, SD:2,103.65; Median: 172.0), the highest per capita operating budget (142.39, SD: 215.925; Median: 103.51),the highest percentage of agencies located in the West region of the US (29.47%, *n* = 112), and the lowest percentage of agencies located in rural areas (11.3%, *n* = 43). In contrast, agencies that did not address mental health had the lowest percentage of agencies with advocacy partnerships (22.7%, *n* = 41), and officers who wear body cameras (61.2%, *n* = 112); the smallest mean number of full time sworn officers (27.47, SD:80.20; Median: 6.99); the smallest percentage of agencies located in the West region of the US (5.46%, *n* = 10), the highest percentage of agencies located in the South region of the US (45.90%, n-84), and the highest percentage of agencies located in a rural county (49.2%, *n* = 90).

Table 2. presents the results from our four logistic regression models predicting different mental health response models within police agencies. Agencies that had a specialized unit to address mental health and that had designated personnel to address mental health had significantly higher odds of having partnerships with advocacy organizations (AOR:1.76, 95%CI: 1.34–2.30; AOR: 1.69, 95%CI: 1.41–2.02), and significantly lower odds of being located in a rural county (AOR: 0.45; 95%CI:0.25, 0.78; AOR: 0.56; 95%CI: 0.45, 0.70). Agencies with specialized units also had significantly higher odds of having more full-time sworn officers (AOR, 1.002; 95%CI: 1.001, 1.003) and use of force complaints per officer (AOR: 2.01, 95%CI: 1.05–3.85), being located in the West region of the US compared to the Northeast (AOR: 3.09; 95% CI: 1.39, 6.83) and being located in a county with a lower percentage of households with an income under the federal poverty limit (AOR:0.95; 95%CI:0.90, 0.99). Agencies that had designated personnel to address mental health and agencies that reported addressing mental health without creating units or designating personnel had significantly fewer full-time sworn officers (AOR: 0.99; 95%CI: 0.99, 0.99; AOR: 0.99; 95%CI: 0.99, 0.99). Agencies that reported addressing mental health without creating units or designating personnel and agencies that did not address mental health had significantly lower odds of having partnerships with advocacy organizations (AOR: 0.72, 95%CI: 0.56, 0.94; AOR:0.35; 95%CI: 0.23, 0.54). Agencies that reported addressing mental health without creating units or designating personnel had significantly higher odds of being located in a rural county (AOR: 1.58; 95%CI: 1.24, 2.01).

**Table 2:** Logistic regression models predicting the availability of different mental health crisis response strategies within US law enforcement agencies (*n* = 2,839), 2020^**T**^

Variable	Agency had specialized unit to address mental health	Agency had designated personnel to address mental health	Addressed mental health, no unit/personnel	Agency did not address mental health
Agency type				
*Sheriff*	*Base Category*	*Base Category*	*Base Category*	*Base Category*
*Local/county police*	0.77 (0.51, 1.15)	0.78 (0.62, 0.99)*	1.31 (0.75, 2.30)	0.93 (0.51, 1.72)
Agency partners with advocacy organizations	1.76 (1.34, 2.30)**	1.69 (1.41, 2.02)**	0.72 (0.56, 0.94)*	0.35 (0.23, 0.54)**
Operating budget per capita (# of residents in census area)	0.99 (0.997–0.999)*	1.00 (0.99, 1.00)	1.001 (0.99, 1.00)	0.99 (0.99, 1.002)
Number of full-time sworn officers	1.002 (1.001–1.003)**	0.99 (0.99, 0.99)**	0.99 (0.994, 0.999)**	0.99 (0.98, 1.00)
# of use of force complaints (per capita)	2.01 (1.05, 3.85)*	1.36 (0.51, 3.60)	0.40 (0.13, 1.23)	1.80 (0.47, 6.88)
Officers wear body cameras	1.09 (0.86, 1.37)	1.12 (0.89, 1.41)	0.86 (0.70, 1.06)	1.19 (0.83, 1.69)
Percent Black in agency’s county	1.01 (0.99, 1.03)	1.00 (0.99, 1.01)	0.98 (0.97, 1.00)	1.01 (0.99, 1.03)
Percent Hispanic in agency’s county	1.01 (0.99, 1.02)	1.00 (0.99, 1.01)	0.99 (0.98, 1.00)	0.98 (0.96, 0.99)
Percent of households with income below the federal poverty limit in agency’s county	0.95 (0.90, 0.99)*	0.99 (0.96, 1.03)	1.00 (0.96, 1.03)	1.02 (0.96, 1.08)
Agency located in rural county	0.45 (0.25, 0.78)**	0.57 (0.46, 0.72)**	1.58 (1.24, 2.01)**	1.20 (0.71, 2.01)
Region				
*Northeast*	*Base Category*	*Base Category*	*Base Category*	*Base Category*
*South*	1.20 (0.56, 2.57)	0.78 (0.58, 1.06)	1.05 (0.70, 1.59)	1.38 (0.86, 2.20)
*Midwest*	1.47 (0.70, 3.09)	0.84 (0.61, 1.17)	0.95 (0.65, 1.38)	0.99 (0.62, 1.58)
*West*	3.09 (1.39, 6.38)**	0.65 (0.44, 0.97)*	0.74 (0.46, 1.20)	0.68 (0.31, 1.51)

^**T**^Table reports AORs with 95% CIs in parentheses

*p < 0.05; **p < 0.01

## Discussion

This study examined the extent to which police agencies nationwide adopt tailored mental health responses, finding that under half of programs designated specific units or personnel towards this purpose. We also identified specific organizational and community-level characteristics associated with having a more specialized model.

Agency size appears to play a role in implementation, as agencies with larger numbers of full-time sworn officers had a higher likelihood of having designated specific units to address mental health. While the effect size was small, specialized units was the only outcome for which agency size was associated with increased odds (AOR > 1), suggesting a relationship between agency size and the presence of specialized mental health units. Trainings on mental health crisis response are resource intensive. For instance, Crisis Intervention Team programs train officers over 40 h of classroom and experiential training on how to recognize a person experiencing a mental health crisis, de-escalate the situation, and connect the person to follow up care (ideally) outside the criminal legal system. Police agencies with more officers may have more capacity to train officers in CIT and ensure their catchment areas have a sufficient number of specialized units or personnel with expertise in mental health crisis response (Compton et al., 2024). More specialized models may also be complicated to implement, as they require police agencies to make changes to multiple aspects of police operations, including patrol, training, scheduling, and dispatch (Watson et al., 2008). As such, larger organizations may be more likely to accommodate these shifts without disrupting other agency activities. Our finding that rural agencies had a lower likelihood of adopting a more specialized mental health crisis response is also notable, as rural agencies tend to be smaller and less resourced compared to agencies located in more urban areas.

We also found having more external partnerships was associated with higher odds of adopting more specialized response models. In recent years, there has been a growing emphasis on developing alternative response models that involve partnerships between police and behavioral health clinicians. Individuals with serious mental illness and their families favor clinician-only response models that include at least one licensed or credentialed behavioral health practitioner, alongside an unlicensed behavioral health practitioner or peer support worker (Pope et al., 2023). A police agency may have a specialized mental health crisis response where a clinician-only response team responds to relevant 911 calls instead of the police, or may adopt on a co-response model (Balfour et al., 2022; SAMHSA, 2025.; Shapiro et al., 2015). While the survey used for the current study does not specifically ask whether agencies have established partnerships with these types of organizations to form a clinician-only or co-response partnership, findings indicate that agencies with established networks beyond agency walls may be best-positioned to move towards these alternative response models. Notably, rural and smaller agencies may not only have fewer resources, but also more limited access to mental health resources and fewer opportunities to form partnerships, which may also constrain their ability to implement specialized mental health crisis responses.

Additionally, we found that agencies located in the West region of the US had a higher likelihood of having designated a specific unit to address mental health. In part, this may reflect regional factors such as higher rates of homelessness, which are higher in cities in the West region of the US (Love & Loh, 2023), and are associated with poor mental health outcomes, including psychiatric distress (Lilanthi Balasuriya et al., 2020). A higher demand for mental health services amongst populations with higher rates of law enforcement encounters may explain why agencies in the West are more likely to adopt a more specialized model of mental health crisis response (Goodison et al., 2020).

Furthermore, agencies in which officers had higher numbers of use of force complaints had a higher likelihood of having adopted a more specialized mental health response. Additionally, agencies in which patrol police wore body cameras had a higher likelihood of having adopted a more specialized response, though this was not significant in our main models. The implications of this are two-fold; first, it suggests that the additional layer of scrutiny brought on by body cameras may be associated with the decision to adopt crisis response models that are widely recognized as improving police mental health crisis response. Indeed, although the evidence is mixed (Taheri, 2016), some evaluations of the CIT model find that CIT implementation results in lower arrest rates of individuals experiencing mental health crises (Cochran et al., 2000; Compton et al., 2014), as well as more frequent linkages to treatment programs (Cochran et al., 2000). Second, it suggests that prior incidents of escalation may be associated with an agency’s decision to develop more formal, specialized responses to mental health crises. In general, although police are often the first responders to mental health crises, findings from qualitative research suggests that many police feel uncomfortable and inadequately trained to respond to mental health or suicidal incidents (Thompson & Kahn, 2018). For example, it is notable that approximately 73% of agencies that reported not formally addressing mental health also reported having written policy or procedural directives on mentally ill persons within their agency. It is likely that officers within these agencies are still responsible for responding to mental health crisis situations but lack specialized training to support them in responding appropriately. A wide body of research indicates that a lack of knowledge and skills related to mental health crisis response may lead police officers to respond with excessive force or neglect to provide proper assistance to an individual experiencing a mental health crisis (Ruiz & Miller, 2004; Ruiz, 1993; Watson et al., 2004). Agencies that have more frequent and documented incidents of escalation may choose to implement specialized models, either driven by officers’ desire for additional training, or external pressures from the public.

This study has several limitations. First, LEMAS data are self-reported, and responses could reflect desirability bias. Second, the outcomes for our four main models came from the survey question “how does the agency address [mental health]”, which itself is vague, as it does not specifically say whether it is referring to mental health referrals, mental health crisis, or decisions around voluntary commitment. As such, due to the vagueness of this question, it is possible that respondents may have interpreted this question in different ways, limiting the reliability of these categories. Specifically, the category ‘Agency addresses mental health but has no designated units or personnel’ is likely interpreted inconsistently across agencies. While some respondents may have been referencing generalized mental health training crisis response training for officers, others may have had internal staff-focused supports (e.g., mental health resources for officers) in mind, or partnerships with external mental health providers (BJS, 2025). Third, while the LEMAS survey asks agencies whether they have partnerships with advocacy organizations, the survey does not specifically ask agencies whether they have partnerships with mental health organizations, which are critical to support mental health crisis response (Seo et al., 2021). As such, the potential partnerships between police agencies and mental health organizations nationally remains an important question for future research. Fourth, as mentioned previously, although clinician only and co-response models are an important models of police mental health crisis response, the LEMAS survey did not ask respondents to report this; as such, the extent to which law enforcement agencies nationwide have adopted these specialized response models remains a gap in knowledge that should be explored further in other studies. Fifth, although our measure of use of force complaints is standardized by the number of full time officers to account for agency size, we did not have access to data on the total number of civilian interactions, the level of forced used, and the outcomes of these encounters, limiting the contextual interpretation of this measure. Sixth, the period under study, 2020, overlaps with the COVID-19 pandemic, in which many police agencies paused non-essential units/programs during the mandated mitigation efforts (Nielson et al., 2022). Finally, although this study uses the most recent LEMAS data from 2020, it is possible that this data is not representative of the country five years later. For example, this study was conducted to widespread implementation of the 988 Suicide & Crisis Lifeline, which began in 2022 (Saunders, 2024). Similarly, since 2020, there have been movement in the public safety and behavioral health landscape, particularly given the developments following the murder of George Floyd by law enforcements. This included increased support for non-police and co-response models, shifts in public attitudes toward law enforcement, and new funding mechanisms.(Anderson & Jorem, 2025; Saunders et al., 2023) These developments may have influenced both the prevalence and the organizational and community predictors of response models, limiting the generalizability of our findings to the current context. Nonetheless, the current study remains valuable for establishing a pre-2020 baseline to inform future research and policy.

## Conclusions

As policies and best-practices related to police responses to mental health related service calls continue to evolve, this study provides the first description of specialized mental health crisis response models within law enforcement agencies nationwide. We found that less than 50% of law enforcement agencies adopted a specialized response that entailed designating specific units or personnel towards mental health crisis activities. Additionally, these results suggest that larger agencies with more ties to community-based organizations had a higher likelihood of having adopted a more specialized response, demonstrating that characteristics of local law enforcement and surrounding environment may contribute to variation in mental health crisis response nationally. Additional research is needed to understand the extent to which police agencies partner with mental health organizations nationwide, and how these partnerships impact the adoption of more specialized response models. Future studies should also assess the prevalence of co-response models within law enforcement agencies nationwide, as well as models which remove police presence from mental health crisis response and instead shift responsibility to mental health providers in crisis situations without safety concerns or criminal activity.

## Appendix

**Table 3:** LEMAS survey questions used in this analysis

Mental health response activities	How did agency address problem/task: Mental health
Agency type	Agency sample type
Operating budget	Agency’s total operating budget for the fiscal year that included December 31, 2020
Agency partners with advocacy organizations	Problem-solving partnership or written agreement with: Advocacy groups
Number of full-time sworn officers	Sum of- Number of Full-time Sworn Officers/Deputies: Female; and Number of Full-time Sworn Officers/Deputies: Male
# of use of force complaints (per capita)	Total number of all use of force complaints in 2020
Officers wear body cameras	Number of video cameras were operated by your agency on a REGULAR basis: On police officers (e.g. body-worn cameras)
